# Ferroptosis in renal cell carcinoma: integrative multi-omics insights and therapeutic perspectives

**DOI:** 10.1097/JS9.0000000000004583

**Published:** 2026-01-13

**Authors:** Xing Wang, Jun Li, Yunfeng Zhang, Ruizhen Huang, Penglin Zhang, Honglin Hu

**Affiliations:** Department of Urology, The Second Affiliated Hospital, Jiangxi Medical College, Nanchang University, Nanchang, China

**Keywords:** ferroptosis, glutathione peroxidase 4 (GPX4), multi-omics integration, renal cell carcinoma (RCC), tumor microenvironment

## Abstract

Renal cell carcinoma (RCC) exhibits marked heterogeneity in its molecular landscape and clinical behavior. Ferroptosis, an iron-dependent and lipid peroxidation-driven form of cell death, has emerged as a biologically relevant process in RCC pathogenesis. This review summarizes recent advances in the multi-omics dissection of ferroptosis in RCC, including findings from genomics, epigenomics, transcriptomics, proteomics, metabolomics, and microbiomics. Key molecular regulators such as VHL, SLC7A11, GPX4, and ACSL4 are highlighted for their roles in ferroptosis sensitivity or resistance. In parallel, insights from single-cell and spatial omics offer new perspectives on cell-type specificity and microenvironmental context. We also discuss the implications of ferroptosis in therapeutic modulation, including potential integration with immune checkpoint inhibitors and metabolic interventions. This review aims to provide a coherent overview of ferroptosis in RCC and inform future mechanistic studies and translational strategies.

## Introduction

Over the past decade, the incidence of kidney cancer has risen steadily, now ranking third among urogenital tumors, with roughly 400 000 new diagnoses and nearly 160 000 deaths worldwide each year^[1]^. Renal cell carcinoma (RCC) is the prevalent histology. Clear cell RCC (ccRCC) accounts for approximately 75% of cases, followed by papillary RCC (pRCC) and chromophobe RCC (chRCC) as the next most common subtypes, with other rarer histological variants comprising the remainder^[2]^. Key molecular hallmarks include pronounced angiogenesis, extensive metabolic reprogramming, and the high frequency of mutations in the von Hippel-Lindau (VHL) gene^[3]^. Systemic regimens such as vascular endothelial growth factor (VEGF) inhibitors and immune checkpoint inhibitors (ICIs) have improved response rates in advanced disease; however, durable outcomes remain limited because of acquired resistance, intratumour heterogeneity, and immune escape^[4]^.

Ferroptosis is a regulated mode of cell death distinct from apoptosis, necrosis, or autophagy. It is driven by iron-dependent lipid peroxidation, defined by the build-up of oxidized polyunsaturated lipids, oxidative stress mediated by Fe^2^⁺, and loss of glutathione peroxidase 4 (GPX4) function, events that collectively collapse the intracellular antioxidant barrier and trigger cell death^[5]^. Growing evidence shows that ferroptosis shapes RCC initiation, progression, and treatment response. Regulators such as ACSL4, FTH1, and HIF2α are often dysregulated; besides modulating iron homeostasis and lipid peroxidation, they correlate with immune cell infiltration, genomic instability, and patient prognosis^[6]^. ACSL4 heightens tumor vulnerability to ferroptosis, whereas overexpression of antioxidant enzymes such as GPX4 enables escape from this death pathway and alters sensitivity to systemic therapies^[7]^. A phenotype resistant to ferroptosis, characterized by low lipid reactive-oxygen species and sustained elevation of GPX4 and SLC7A11 (also known as xCT), is frequently observed in tumors that fail antiangiogenic or immunotherapy treatments^[8]^.

**HIGHLIGHTS**

- Multi-omics research identifies ferroptosis as a key process shaping renal cell carcinoma (RCC) progression and therapy response.
- Genetic, epigenetic, and proteomic alterations yield potential biomarkers for prognosis and patient stratification.
- Ferroptosis influences tumor immunity, metabolism, and drug resistance, creating perioperative and therapeutic opportunities.
- Core regulators such as glutathione peroxidase 4 and ACSL4 serve as promising intervention targets.
- Integrating ferroptosis insights with surgical and precision oncology strategies may guide future RCC management.

**Figure 1.**
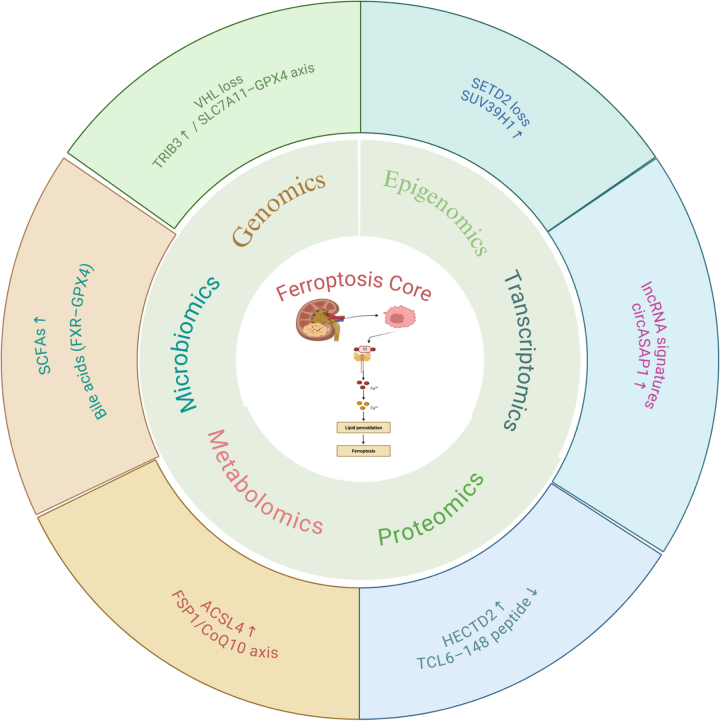
Multi-omics regulatory landscape of ferroptosis in renal cell carcinoma (RCC).

The regulatory network governing ferroptosis is highly complex, spanning genetic alterations, epigenetic marks, transcriptional control, metabolic rewiring, and cues from the tumor microenvironment. Public multiomics resources such as TCGA, CPTAC, and FerrDb now allow systematic mapping of this landscape^[9]^. Genomic, epigenomic, transcriptomic, proteomic, and metabolomic technologies have already clarified cancer heterogeneity and intricate signaling loops, providing fresh avenues to interrogate ferroptosis^[10,11]^. Although many studies have examined ferroptosis in RCC, most focus on single omic layers or isolated pathways, leaving a full multiomics synthesis lacking. This review, therefore, collates current progress from an integrated perspective. We summarize the contributions and interactions of genomic, epigenomic, transcriptomic, proteomic, metabolomic, and immunological dimensions to ferroptosis control, and discuss the impact of the tumor microenvironment, advances in artificial-intelligence analytics, emerging therapeutic strategies, and future research priorities (Fig. 1). Review methodology: A structured literature search was performed across PubMed, Web of Science, and Scopus using the terms “ferroptosis,” “RCC,” “kidney cancer,” “ferroptosis regulator,” “GPX4,” “SLC7A11,” and their Boolean combinations. The search covered publications from January 2013 to August 2025, corresponding to the decade in which ferroptosis became an established research focus. The initial search yielded 1204 records. After removal of duplicates, titles and abstracts were screened to exclude studies unrelated to RCC, lacking ferroptosis relevance, limited to case descriptions, or without molecular or mechanistic data, resulting in the exclusion of 742 articles. Full-text assessment was then conducted on the remaining literature. Articles were evaluated for their relevance to core ferroptosis pathways (e.g., GPX4, SLC7A11, ACSL4, and FSP1), multi-omics regulatory interactions, subtype-specific ferroptosis vulnerabilities, and potential translational or perioperative clinical implications. A further 332 studies were excluded during this stage due to insufficient mechanistic depth, lack of multi-omics evidence, or absence of clinically meaningful insights. In total, 130 publications met the predefined inclusion criteria and were incorporated into the final synthesis, forming the evidentiary foundation for the ferroptosis regulatory networks, multi-omics mechanisms, and emerging surgical and clinical implications discussed in this review. Review framework and reporting: This work is an integrative narrative review, not a systematic or scoping review; consequently, a PRISMA/PRISMA-ScR flow diagram is not applicable. In line with good-practice guidance for narrative reviews, we explicitly define our scope, report a transparent search strategy and selection principles, and synthesize evidence qualitatively across omics domains. AI transparency statement: No generative AI tools were used for conception, literature search, data extraction, figure creation, writing, or editing in this manuscript; we cite the TITAN-AI 2025 transparency principles solely to document adherence to reporting norms, which are orthogonal to literature-review reporting standards^[12]^. Our goal is to offer a conceptual and methodological scaffold to spur mechanistic discovery and guide the development of targeted interventions.

This integrative diagram summarizes the major genomic, epigenomic, transcriptomic, proteomic, metabolomic, and microbiome-derived mechanisms that converge on the ferroptosis machinery in RCC. Genomic alterations such as VHL loss and TRIB3/SLC7A11-GPX4 pathway activation modulate lipid-peroxidation sensitivity. Epigenomic regulators, including SETD2 and SUV39H1, influence chromatin accessibility and ferroptosis-related transcriptional output. Transcriptomic programs involve ferroptosis-associated lncRNAs and circular RNAs (e.g., circASSAP1), which fine-tune xCT/GPX4 axis activity. Proteomic features, including HECTD2 and the TCL6-148 peptide, reshape protein-level redox buffering. Metabolomic pathways such as FSP1/CoQ10 activity, ACSL4 upregulation, and lipid remodeling determine susceptibility to iron-dependent lipid peroxidation. Microbiome-derived metabolites – including short-chain fatty acids (SCFAs) and bile acids – further modulate ferroptosis via systemic effects on iron handling and lipid metabolism. Together, these multi-layered regulatory networks define the ferroptosis phenotype of RCC and its potential relevance for precision oncology.

## Genomics

Large chromosomal shifts dominate the ferroptosis landscape in ccRCC. A TCGA survey of 57 ferroptosis-related genes (FRGs) separated tumors into three CNA-driven patterns; the group with 5q and 8q gains plus 3p loss showed the highest “ferroptosis score,” an elevated mutation burden, and the poorest survival^[13]^. Across four RCC subtypes, the same dataset revealed widespread dosage increases for the cystine antiporter SLC7A11 and reductions for antioxidant genes MT1G and HMOX1, underscoring aneuploidy – rather than point mutation – as the primary genomic lever that tilts tumors away from lethal lipid peroxidation^[14]^.

Among focal amplifications, the mitotic kinesin KIF23 (15q23) stands out. Copy gain occurs in roughly one in eight ccRCCs and accompanies higher T stage, greater neoantigen load, and a transcriptional programme that suppresses ferroptosis^[15]^. Such dosage-driven cell-cycle acceleration may buffer dividing cells from oxidative damage by sustaining glutathione (GSH) and lipid-repair pathways.

Gene amplification sometimes works through proteostasis. The E3 ligase COP1 is gained or overexpressed in about a third of tumors; excess COP1 tags the pro-ferroptotic enzyme ACSL4 for degradation, lowering ACSL4 protein by roughly 70% and sharply reducing erastin-induced cell death^[16]^. A similar logic applies to the pseudokinase TRIB3. High gene copy or mRNA level of TRIB3 preserves the SLC7A11/GPX4 axis and underlies resistance to the tyrosine-kinase inhibitor sunitinib; silencing the amplified allele restores lipid-ROS accumulation and resensitizes tumors^[17]^.

Loss-of-function events, though less common, can be equally decisive. Frameshift mutations that truncate the histone demethylase KDM5C occur in approximately 18% of male ccRCCs. The resulting clones accumulate glycogen, channel flux through the pentose-phosphate pathway, and boost NADPH/GSH, all of which blunt ferroptosis^[18]^. In an iron-driven renal-cancer model, BRCA1 haploinsufficiency doubled total amplified chromosomal length, and simultaneously suppressed ACSL4 and COX-2, suggesting that faulty DNA repair fosters CNA patterns favoring ferroptosis escape^[19]^.

Not every dosage change protects the cell. Up-regulation of the GSH-degrading enzyme CHAC1 in advanced tumors drains intracellular GSH, raises lipid peroxides, and predicts shorter survival, placing CHAC1 among the few genomic lesions that actively sensitize RCC to ferroptosis^[20]^. Co-expression and CNA analysis have also highlighted PROM2 and PLIN2 as lipid-handling hubs whose high genomic dosage tracks with diminished ferroptotic activity and poorer outcome, broadening the roster of dosage-dependent modulators beyond classic iron or cystine transporters^[21]^.

Together, these findings paint a genome shaped more by broad aneuploidy and focal copy shifts than by recurrent single-nucleotide hits. Amplifications that curb lipid peroxidation (KIF23, COP1, TRIB3, and PROM2), truncations that distort redox metabolism (KDM5C and BRCA1), and the occasional pro-ferroptotic gain (CHAC1) weave a DNA-level tapestry.

## Epigenomics

While the genome fixes the DNA code, the epigenome overlays reversible chemical and structural tags that dictate which parts of that code are read, thereby tuning a tumor’s threshold for ferroptotic death.

In ccRCC, one of the most influential tags is H3K9 trimethylation. When the methyl-transferase SUV39H1 is abundant, a dense H3K9me3 cap sits on the DPP4 promoter, keeping NOX1 activity muted and lipid peroxides in check; genetic knockdown or chaetocin treatment strips away this cap, iron floods the cell, and xenografts collapse under a surge of ferroptosis^[22]^. A different histone, H3K14, tells the opposite story: loss of the acetyl-transferase KAT7 erodes H3K14ac, accelerates S-phase, and paradoxically blunts erastin sensitivity, whereas KAT7 restoration reins in proliferation and reopens the ferroptotic gate^[23]^. Pivot again to H3K36 – here, dropping the trimethyl-writer SETD2 drains H3K36me3 from the FECH locus, starves heme synthesis, liberates Fe^2^⁺, and renders ccRCC cells hyper-susceptible to erastin^[24]^. Three marks, three enzymes, and three very different ferroptosis outcomes.

Epigenetic programs recalibrate ferroptosis thresholds in RCC. At the chromatin level, H3K9 trimethylation by SUV39H1 deposits a repressive cap on the DPP4 promoter to dampen NOX1 activity and lipid peroxidation; genetic knockdown or pharmacologic inhibition removes this mark and unleashes ferroptotic death *in vivo*^[25]^. Conversely, loss of the acetyl-transferase KAT7 reduces H3K14ac, accelerates S-phase, and blunts erastin sensitivity, whereas KAT7 restoration re-opens the ferroptotic gate^[25]^. SETD2-dependent H3K36me3 at the FECH locus supports heme biosynthesis and limits labile iron; SETD2 loss drains H3K36me3, elevates Fe2^+^, and renders ccRCC cells hyper-susceptible to erastin. Parallel RNA-layer control further tunes antioxidant capacity: truncating KDM5C rewires carbon flux toward the pentose-phosphate pathway to boost NADPH/GSH and ferroptosis resistance, whereas restoring KDM5C or blocking glycogenolysis re-sensitizes tumors^[26]^. MicroRNA circuits can lower the GPX4 barrier – e.g., miR-324-3p-mediated GPX4 repression increases ferroptotic vulnerability in RCC cells^[27]^. Collectively, these epigenetic nodes nominate biomarker candidates and sensitizers to xCT/GPX4-directed strategies and justify incorporation into biomarker-guided trials in RCC.

Beyond covalent marks, chromatin architecture itself can make or break lipid-ROS death. Truncating mutations in the X-escape demethylase KDM5C (seen in roughly one-fifth of male tumors) dial up H3K4me3 at glycogen and pentose-phosphate genes, raising NADPH/GSH buffers that neutralize peroxides^[18]^. In renal medullary carcinoma, loss of the SWI/SNF core subunit SMARCB1 flips a TFCP2L1-to-MYC switch; adding SMARCB1 back restores lipid oxidation and kills the cells^[28]^. Translocation RCC takes a third route: oncogenic TFE3 fusions occupy thousands of H3K27-acetylated enhancers, synchronously driving oxidative phosphorylation and a ferroptosis-resistant transcriptome – CRISPR deletion of the fusion abruptly breaks that spell^[29]^.

RNA modifications overlay yet another layer. A five-gene m6A-ferroptosis score – woven from writers, readers, and erasers – tracks with immune checkpoint expression and bleak survival^[30]^, while a six-lncRNA ceRNA web (notably PVT1→miR-axes→SLC7A11) carves out a high-risk epigenetic niche that pairs immune evasion with ferroptosis escape^[31]^. Together, these methyl-coded circuits fine-tune the very transcripts that the chromatin landscape releases.

Taken as a whole, renal tumors do not rely on a single hierarchical epigenetic script; instead, they mix and match histone writers, erasers, remodelers, and RNA codes to steer ferroptosis toward either vulnerability or resistance. Mapping these intertwined layers is now yielding multiple entry points – from chaetocin to m6A inhibitors – for rekindling lipid-peroxide lethality in refractory disease.

## Transcriptomics

**Figure 2.**
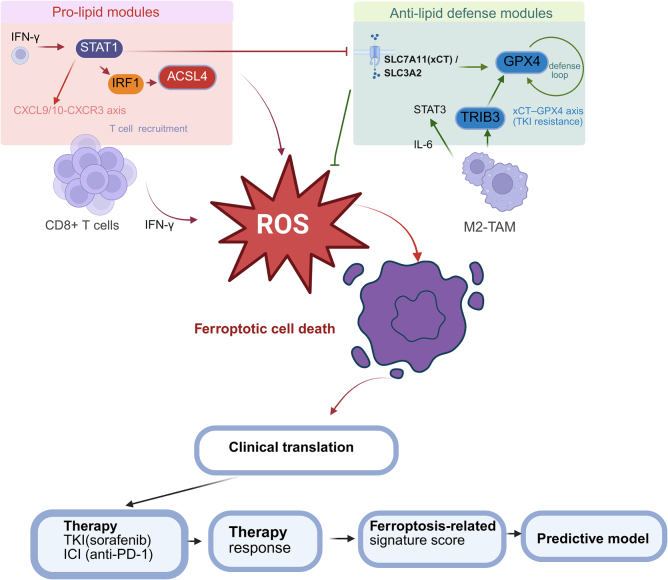
Simplified overview of ferroptosis regulation in renal cell carcinoma based on transcriptomics and immune modulation.

Bulk RNA-seq analyses point to ferroptosis wiring as a strong determinant of outcome in renal cancer (Fig. 2). Early long-non-coding RNA (lncRNA) panels laid the groundwork: a nine-lncRNA score separated ccRCC into immune-inflamed and immune-silent phenotypes with a 3-year AUC of 0.78^[32,33]^; a seven-lncRNA update reproduced the same T-cell activation gradient across three external datasets^[34]^. Subsequent five-and eight-lncRNA models sharpened risk strata, isolating hypoxia-rich, cytokine-high tumors that also show lower predicted IC_50_ values for kinase inhibitors such as sunitinib and dasatinib^[35,36]^. Despite dissimilar gene rosters, high-risk groups consistently display reduced cystine import and up-regulated checkpoint molecules – molecular hallmarks of ferroptosis escape and immunosuppression.

This figure presents a transcriptomics-based schematic of ferroptosis regulation in renal cell carcinoma (RCC). It highlights the key molecular modules involved in ferroptosis, including pro-lipid and anti-lipid defense pathways. Pro-lipid modules, activated by IFN-γ and STAT1, lead to lipid peroxidation and ROS generation, promoting ferroptotic cell death, with immune cell recruitment through the CXCL9/10-CXCR3 axis. Anti-lipid defense modules, such as GPX4, protect against lipid peroxidation and ferroptosis, with the xCT-GPX4 axis contributing to resistance against tyrosine kinase inhibitors (TKIs). The tumor microenvironment and immune cells, particularly M2 macrophages, modulate these processes, influencing ferroptotic susceptibility. The diagram also emphasizes the potential clinical applications of these insights, including combining TKI and immune checkpoint inhibitors (ICIs) to enhance therapeutic response and develop predictive models based on ferroptosis-related biomarkers.

mRNA-based signatures bring metabolic nuance. A six-gene score built around DPEP1 and ACADSB stratifies survival independently of stage and tumor-mutation burden^[37,38]^, whereas a nine-gene model featuring the glutamine-metabolism regulator GLS2 links high risk to reductive NADPH flux and resistance to lipid-peroxide stress^[39]^.

Single-gene studies explain why patients with similar composite scores can diverge clinically. Low expression of the ferritinophagy adaptor NCOA4 predicts sparse CD8⁺infiltration and poor survival ^[40,41]^. Elevated LPCAT3 remodels phospholipids, intensifies ER-stress-coupled ferroptosis, and paradoxically associates with better outcome – possibly via enhanced tumor immunogenicity^[42,43]^. Up-regulated FDFT1 suppresses AKT yet raises lipid-ROS^[44]^; conversely, metallothionein MT1G buffers GSH to blunt ferroptotic damage^[43]^.

Regulatory RNAs complete the circuit. lncRNA ZFAS1 heightens mitochondrial iron flux through a miR-185-5p→SLC25A28 loop^[45,46]^ (Tao 2025; Xu 2023), while circASAP1 binds HNRNPC and destabilizes GPX4, defining a circRNA-high risk arm^[47]^. At the microRNA tier, endogenous miR-4735-3p blocks iron export via SLC40A1, whereas therapy-induced miR-324-3p silences GPX4; both raise lipid-derived ROS^[27,48]^.

Bulk profiles average millions of cells; single-cell and spatial assays show which cells drive ferroptosis programmes and where they sit in the tumor.

In ccRCC, scRNA-seq of seven tumors revealed macrophage and endothelial subsets co-expressing PDK4 and AMN; their abundance outperforms stage as a survival predictor^[49]^. Spatial maps of the same specimens identified MIOX-bright epithelial islands encircled by exhausted CD8⁺T cells – niches invisible in bulk data^[47]^. pRCC highlights clinical value: projecting a bulk nine-lncRNA score onto scRNA data uncovered fibroblast-rich and pericyte-dense pockets in high-risk tumors, regions tied to kinase-inhibitor resistance^[50,51]^. Such cell-type precision is now guiding micro-environment-centered ferroptosis strategies. By anchoring molecular signatures in spatial context, single-cell technologies reveal MIOX-high clones, ROS-primed myeloid cells, and CAF pockets – micro-domains where precision ferroptosis interventions may achieve maximal impact.

## Proteomics

Proteomic platforms such as affinity purification, quantitative mass spectrometry, and mapping of post-translational modifications have started to chart ferroptosis control in renal carcinoma at the protein level. A GFP-tag pull-down showed that the tumor-suppressor BAP1 binds HAT1 together with the entire COPI coatomer, a finding that connects histone acetylation and vesicle traffic to lipid-peroxide sensitivity in clear-cell RCC^[52]^.

Ubiquitin circuits create the baseline for ferroptosis. The E3 ligase HECTD2 is frequently amplified. Either shRNA silencing or pharmacologic inhibition with veratric acid destabilizes HECTD2, lowers SLC7A11 and GPX4, increases ferrous iron and malondialdehyde, and limits xenograft growth^[53]^. In contrast, the de-ubiquitylase USP35 stabilizes IAP proteins and NRF2. Knockdown of USP35 dismantles both apoptosis and ferroptosis barriers and improves drug response^[54]^. A third switch, the adaptor protein TRIB3, acts through its abundance rather than catalytic activity: depletion reduces SLC7A11 and GPX4 and restores sensitivity to sunitinib^[17]^.

Metabolic enzymes and micro-peptides adjust the speed of execution. A 39 amino-acid peptide derived from lncRNA *TCL6* (TCL6148) binds GOT1, suppresses GPX4, and doubles the tumor-shrinkage achieved by sunitinib^[55]^. A phytochemical screen of more than 1000 compounds identified five molecules that up-regulate the steroidogenic enzyme CYP11A1, reduce cholesterol, and curtail migration by promoting ferroptosis^[56]^. Lipid catabolism offers an additional lever. The FAAH inhibitor URB597 works with the GPX4 blocker RSL3 to raise 4-HNE and curb PI3K–AKT signaling^[57]^. The natural bibenzyl moscatilin produces a similar outcome by lowering phosphorylated JAK2 and STAT3 while suppressing xCT and increasing intracellular iron^[58]^.

Stress-response proteins decide whether the cell commits to ferroptosis. Silencing the redox regulator APEX1 releases APP, activates p53, suppresses xCT, and drives iron overload, a sequence that pushes cells into ferroptosis and reduces invasiveness^[59]^. Conversely, the zinc-finger transcription factor ZNF83 amplifies NRF2 target genes and shields tumors from erastin. CRISPR deletion of ZNF83 reverses this protection and restricts xenograft growth^[60]^. Extracellular signaling also plays a role. Reduced levels of the cytokine GDF15 lower lipid-ROS and predict poor outcome, whereas re-expressing GDF15 restores ferroptotic markers^[61]^.

These investigations depict a hierarchical control scheme in which ubiquitin modifiers act as gatekeepers, metabolic switches modulate the tempo, and stress sensors deliver the final verdict. Therapeutic intervention at nodes such as HECTD2, USP35, or TCL6148 may therefore reopen ferroptotic pathways in treatment-refractory renal cancer.

## Metabolomics

Metabolomic surveys based on isotope tracing and high-resolution mass spectrometry show that clear-cell ccRCC relies on a lipid-rich, iron-laden biochemistry that both fuels growth and shapes vulnerability to ferroptosis. Hypoxia-induced lipid desaturases, up-regulated through HILPDA signaling, expand the pool of poly-unsaturated acyl chains, and early tumors accumulate readily exchangeable iron; once the glutathione–GPX4 defence falters, iron catalyzes rapid lipid peroxidation and cell death^[62,63]^. Histological mapping of more than 1000 surgical cores confirms abundant iron in low-grade disease, with a progressive decline as lesions dedifferentiate or metastasize, suggesting deliberate iron restriction as an evasion mechanism^[64]^.

A first regulatory layer involves fatty-acyl activation. Diminished expression of ACSL4 is associated with shorter survival, whereas either re-expression of ACSL4 or transcriptional activation by GABPA enlarges the PUFA-CoA pool and heightens lipid damage^[65,66]^. By contrast, ACSL3 directs exogenous fatty acids into neutral lipid droplets; knockout curtails tumor growth yet simultaneously reduces ferroptosis sensitivity by depleting oxidizable substrate^[67]^. Fatty-acid catabolism provides complementary switches. Lower activity of the dehydrogenase ACADSB and higher expression of the thioesterase ACOT8 both coincide with the advanced stage and weak lipid-ROS signatures, highlighting β-oxidation as a brake on ferroptosis^[68,69]^. Mitochondrial redox regulation reinforces this scheme: loss of MDH2 stabilizes membrane oxidoreductase FSP1 and suppresses peroxidation, whereas reintroducing MDH2 reverses these changes^[70]^. Inhibition of the iron-sulphur scaffold ISCA2 produces the opposite effect, raising labile iron, lowering HIF translation, and driving VHL-deficient tumors into ferroptosis^[71]^.

Systemic and micro-environmental metabolites modulate the same pathway. The adipokine chemerin, elevated in obesity, represses fatty-acid oxidation; tumors exposed to high chemerin store more neutral lipid and resist peroxidative stress, a phenotype reversed by silencing chemerin or its receptors GPR1 and CMKLR1^[72,73]^. Tumors also import antioxidants directly: glycosaminoglycan-mediated uptake of LDL and HDL delivers vitamin E that quenches peroxidized lipids; enzymatic removal of glycosaminoglycans restores ferroptotic sensitivity^[74]^. Cholesterol acts as a second exogenous buffer; its accumulation suppresses lipid-ROS, whereas statin therapy or LDL-receptor depletion reactivates lethal peroxidation^[75]^.

Emerging therapeutic designs exploit these metabolic weak points. An acid-sensitive metal–organic framework MIL-101 (Fe)co-loaded with ferrous ions and the GPX4 inhibitor RSL3 releases both cargos within the tumor, producing a sustained surge of lipid peroxides that eradicates orthotopic ccRCC with limited off-target toxicity^[76]^. In a complementary approach, inhibition of FAAH by URB597 elevates oxidized lipids and synergizes with RSL3 to silence PI3K and AKT signaling, converting metabolic stress into tumor regression^[57]^.

**Figure 3.**
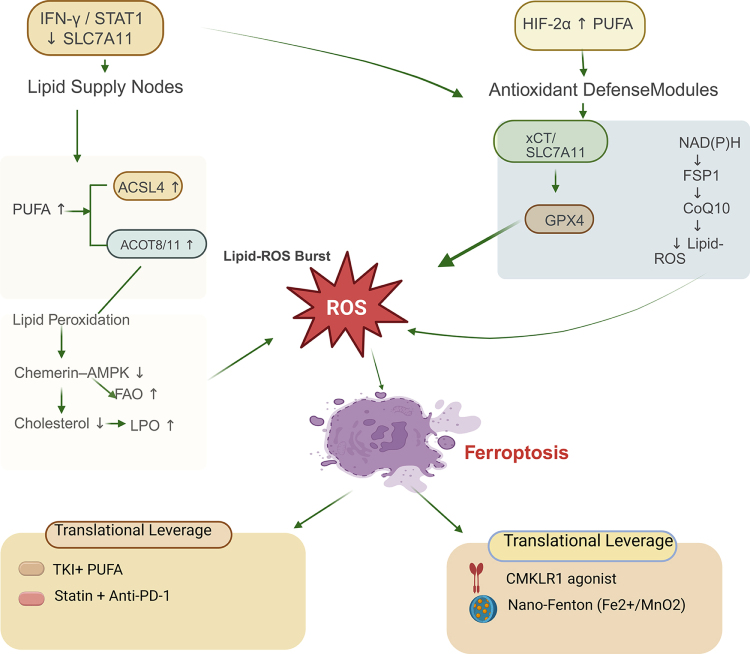
Metabolic vulnerabilities and therapeutic modulation of ferroptosis in RCC.

Collectively, these findings depict ferroptosis control as a metabolic ledger: enzymes such as ACSL4, ACADSB, MDH2, and ISCA2 add pro-oxidant entries, whereas lipid droplets, vitamin E import, cholesterol buffering, and chemerin signaling populate the antioxidant column. Therapies that tip this balance – by depleting sterols, blocking antioxidant uptake, or reinstating iron flux – may turn the metabolic signature of ccRCC from a survival advantage into a therapeutic liability (Fig. 3).

This diagram illustrates the role of metabolic vulnerabilities in RCC in regulating ferroptosis and highlights potential therapeutic strategies for modulation. Lipid supply nodes, activated by IFN-γ and STAT1, promote the accumulation of polyunsaturated fatty acids (PUFAs) and lipid peroxidation, which lead to ferroptosis through lipid-ROS burst. Key enzymes such as ACSL4 and ACOT8/11 are pivotal in lipid metabolism, driving this process. On the other hand, antioxidant defense mechanisms, including GPX4 and the xCT/SLC7A11 axis, counteract lipid ROS accumulation and protect cells from ferroptosis. The figure further highlights therapeutic approaches targeting these metabolic pathways, such as combining tyrosine kinase inhibitors (TKI) with PUFAs, statins with anti-PD-1 therapy, and agents like CMKLR1 agonists and Nano-Fenton (Fe^2+^/MnO_²_), which enhance ferroptosis by modulating lipid metabolism and redox balance. These insights underline the therapeutic potential of targeting metabolic vulnerabilities to promote ferroptosis in RCC.

## Microbiomics

Microbiomics – often designated metagenomics – unites next-generation sequencing with metabolomic and culturomic pipelines to chart bacteria, fungi, viruses, and archaea, together with their functional read-outs, in compartments that range from the intestinal lumen to renal-tumor stroma^[77,78]^. These multilayered datasets have revealed subtle host–microbe dialogues regulating metabolism, chromatin dynamics, and immune tone – processes tightly coupled to lipid peroxidation and ferroptosis^[79]^. Yet an explicit microbiomic view of ferroptosis in RCC remains rare; most links derive from extrapolation or small single-center cohorts, underscoring a conspicuous gap in the RCC canon^[80]^.

RCC-relevant microbiomics and ferroptosis. Multiple cohorts now detect low-biomass intratumoral microbiota in RCC and link microbial burden/composition to prognosis and immunotherapy benefit^[80,81]^. Mechanistically, gut- and tumor-derived metabolites shape ferroptosis gates: short-chain fatty acids (e.g., butyrate) can sensitize cells to ferroptosis, whereas bile acids frequently suppress lipid peroxidation by activating FXR and upregulating GPX4/FSP1 programs^[79,82]^. Together, these data nominate microbiome composition and metabolite signatures as biomarker candidates and modifiable levers (diet/probiotics/FMT) to tune ferroptosis vulnerability in RCC – while emphasizing the need for RCC-specific, prospective validation. From a clinical translation standpoint, RCC-specific, prospective trials of microbiome-modulating strategies remain limited; most interventional evidence to date derives from broader oncology settings of immunotherapy. Embedding longitudinal assays of ferroptosis biomarkers (e.g., 4-HNE/MDA, GPX4/FSP1, and lipid peroxidation imaging) together with microbial metabolites in RCC cohorts receiving IO/TKI combinations would provide disease-specific validation and clarify causality.

Microbial metabolites act as chemical rheostats. Short-chain fatty acids (SCFAs), secondary bile acids, and indole derivatives converge on histone deacetylases, NRF2, and the SLC7A11–GPX4 antioxidant circuit, thereby modulating ferroptotic thresholds^[25,83]^. In clear-cell RCC, multi-omic layers reveal that elevated SCFA signatures track with diminished GPX4 expression in VHL-mutant clones, hinting at a metabolite–gene synergy awaiting experimental dissection^[84]^. Population-scale Mendelian-randomization further links pro-inflammatory microbial taxa to heightened RCC risk through cytokine-driven ferroptotic stress^[85]^.

Low-biomass bacterial and fungal consortia are now detectable within ccRCC specimens; their α-diversity inversely correlates with survival and aligns with ferroptosis-associated immune phenotypes^[81,86]^. Two broad patterns emerge: tumors rich in lipid-peroxidizing microbes exhibit high ferroptosis-gene scores, whereas those dominated by antioxidant commensals do not^[87]^. Systemically, gut enterotypes enriched in *Akkermansia* or *Ruminococcaceae* correlate with superior checkpoint-blockade responses, plausibly via ferroptosis-primed antigenicity and invigorated CD8⁺infiltration; antibiotic exposure erodes these consortia and blunts both microbial diversity and ferroptosis-linked danger signaling^[88,89]^.

Nutritional strategies – whether fiber-laden Mediterranean patterns or ketogenic regimens – alongside targeted probiotics, fecal microbiota transfer, and precision antibiotics are being explored as levers to recalibrate microbial communities and, by extension, tumor-ferroptosis thresholds^[90]^. Parallel drug-development programs now exploit nanocarriers and phytochemicals to trigger ferroptosis selectively in malignant cells while sparing normal parenchyma^[91]^. Robust biomarkers that couple dysbiosis indices with ferroptosis-gene signatures will be pivotal – provided the current paucity of microbiomics-driven RCC data is addressed through well-powered, multi-center studies.

## Tumor micro-environment and immunomic crosstalk

Within the Cancer Genome Atlas cohort, high SLC7A11 transcript levels coincide with PD-L1 up-regulation, regulatory T-cell build-up, and an M2-skewed macrophage compartment, pointing to an antioxidant defense that underwrites local immunosuppression^[92]^. Conversely, tumors that secrete abundant CX3CL1 display stronger lipid peroxidation, attract CD8⁺lymphocytes, and yield longer survival, implying that pro-ferroptotic pressure favors effector recruitment^[93]^.

Extending to spatial resolution, multi-region metabolomic-transcriptomic maps trace oxidative islands rich in polyunsaturated lipids where dendritic cells and cytotoxic T cells cluster, whereas glycolytic territories remain immunologically sparse^[94]^. After neoadjuvant tyrosine-kinase inhibition, single-cell sequencing detects an IL-6–JAK2–STAT3–SLC7A11 clone that regrows the lesion; knocking down SLC7A11 in this subset reinstates drug sensitivity and revives interferon signaling^[95]^. A similar pattern emerges in renal medullary carcinoma: re-expression of SMARCB1 dismantles a MYC-driven antioxidant programme and opens the stroma to neutrophils and T cells^[96]^.

At the systemic level, obesity-linked chemerin suppresses fatty-acid oxidation, enlarges neutral-lipid stores, and curtails ferroptosis; silencing chemerin or blocking its receptors GPR1 and CMKLR1 reverses these metabolic changes and enhances natural-killer-cell infiltration^[72,97]^. Moreover, tumor-surface glycosaminoglycans capture LDL and HDL particles that ferry vitamin E into cells, directly quenching peroxidized phospholipids; enzymatic removal of these glycans restores ferroptotic sensitivity and boosts CD8⁺activity^[74]^. Cholesterol serves as a second extracellular buffer: its accumulation lowers membrane susceptibility to oxidation, whereas statins or LDL-receptor depletion reinstate lethal peroxidation and improve checkpoint blockade efficacy^[75]^.

Clinically, an eight-gene panel that combines lipid-peroxidation drivers with cytolytic markers stratifies patients for immunotherapy more accurately than either feature alone. Tumors bearing active ferroptosis signatures and dense effector infiltrates benefit most from PD-1 blockade, whereas antioxidant-rich, immune-silent lesions remain refractory^[98]^. Together, these findings argue that dismantling metabolic shields – by limiting antioxidant import, disrupting lipid storage, or modulating iron balance – could amplify immune clearance in renal cancer (Table 1).

**Table 1:** Agents that induce or sensitize renal-cell carcinoma to ferroptosis.

–	Agent/formulation	Class/translational angle	Principal mechanism of ferroptosis induction	Key RCC model (s)	Study type	Evidence Level	Translational readiness	References
A Clinically deployed targeted drugs repurposed for ferroptosis								
1	Sorafenib	VEGF/RAF TKI (approved, adjuvant FI)	Inhibits system Xc- and depletes GSH	786-O, Caki-1; patients	cell + xeno + clin	Clinical	Practice-adjacent	^[99]^
2	Everolimus	mTOR inhibitor (approved, FI sensitizer)	4EBP1 blockade lowers GPX4, synergizes with erastin/RSL3	769-P, ACHN	cell + xeno	Preclinical (*in vivo*)	Early clinical potential	^[100]^
B Classical chemical inducers and pathway-targeted sensitizers								
3	Erastin	System Xc- inhibitor (tool)	Cystine starvation → GSH loss	786-O, Caki-1	cell + xeno	Preclinical (*in vivo*)	Exploratory	^[100]^
4	RSL3	Covalent GPX4 inhibitor	Unchecked lipid-peroxide accrual	ACHN, 786-O	cell + xeno	Preclinical (*in vivo*)	Exploratory	^[100]^
5	DPP9 inhibitor (Val-boroPro)	NRF2/SLC7A11 axis blocker	Destabilizes NRF2, reverses sorafenib resistance	a	cell	*In vitro*	Exploratory	^[96]^
6	STAT3 inhibitor (Stattic)	IL-6–STAT3 escape blocker	Prevents SLC7A11 up-regulation after TKI	/	cell	*In vitro*	Exploratory	^[101]^
7	USP8 inhibitor	GPX4 degrader, immuno-sensitizer	Promotes GPX4 ubiquitination; boosts anti-PD-1 efficacy	murine RCC	cell + xeno	Preclinical (*in vivo*)	Emerging	^[20]^
8	FSP1 degrader (PROTAC)	CoQ10 shield remover	Re-sensitizes MDH2-low tumors	/	cell	*In vitro*	Exploratory	^[102]^
C Natural products and dietary phytochemicals								
9	Artesunate	Semi-synthetic artemisinin	Blocks system Xc-, ROS burst	786-O, A498	cell + xeno	Preclinical (*in vivo*)	Emerging	^[3]^
10	Lycorine	Amaryllidaceae alkaloid	↑ ACSL4, ↓ GPX4 → lipid-ROS	786-O, ACHN	cell	*In vitro*	Exploratory	^[16]^
11	Curcumin (+ sunitinib)	Polyphenol, TKI re-sensitizer	Up-regulates ADAMTS18, suppresses SLC7A11	769-P, 786-O	cell + xeno	Preclinical (*in vivo*)	Emerging	^[11]^
12	Luteolin	Flavonoid, HO-1 agonist	HO-1-mediated Fe^2^⁺ surge, lipid peroxidation	786-O, Caki-1	cell + xeno	Preclinical (*in vivo*)	Emerging	^[12]^
13	Ursolic acid (+sorafenib)	Triterpenoid	Degrades MCL-1 and SLC7A11	786-O	cell + xeno	Preclinical (*in vivo*)	Emerging	^[19]^
14	2-Undecanone	Plant volatile	STAT3→GPX4 inhibition, GSH loss	/	cell + xeno	Preclinical (*in vivo*)	Emerging	^[15]^
15	Icariside II	Prenylated flavonoid	miR-324-3p-mediated GPX4 repression	A498, 786-O	cell + xeno	Preclinical (*in vivo*)	Emerging	^[45]^
16	Ginsenoside Rh4	Ginseng saponin	Inhibits NRF2; ↓ GPX4/SOD1/CAT	Caki-1, 786-O	cell	*In vitro*	Exploratory	^[13]^
17	JS-K	NO-releasing pro-drug	GSH depletion; c-Myc→GSTP1 suppression	786-O	cell + xeno	Preclinical (*in vivo*)	Emerging	^[14]^
D Metabolic-enzyme and mitochondrial leverage								
18	Cyst (e) inase (+rapamycin)	Enzymatic cystine depletion	Starves cystine; drives FI in FH-deficient RCC	UOK262; xeno	cell + xeno	Preclinical (*in vivo*)	Emerging	^[103]^
19	α-NETA (GPR1/CMKLR1 blocker)	Lipid-droplet disruption	Collapses neutral-lipid storage, dual apoptosis + FI	786-O; xeno	cell + xeno	Preclinical (*in vivo*)	Emerging	^[70]^
E Nanotechnology and physical platforms	A							
20	MIL-101(Fe)@RSL3	pH-responsive MOF nanodrug	Co-releases Fe^2^⁺ + RSL3 (“waterfall” ROS)	Patient-derived ccRCC; xeno	cell + xeno	Preclinical (*in vivo*)	Emerging	^[73]^
21	Fe₃O₄-PEG-PLGA + mild heat	Thermo-responsive nano-generator	45°C heat releases Fe^2^⁺, ↓ SLC7A11	Orthotopic ccRCC	cell + xeno	Preclinical (*in vivo*)	Emerging	^[104]^
22	Fe/Mn dual nano-catalyst	ROS-amplifying nano-platform	Fenton-like Fe/Mn catalysis, lipid peroxidation	/	cell	Preclinical (*in vivo*)	Emerging	^[8]^

**Study type**: cell = *in vitro*; xeno = mouse xenograft; clin = clinical/patient data.

**Evidence level**: *In vitro* = demonstrated only in cell-based experiments; Preclinical (*in vivo*) = supported by animal models; Clinical = validated in patient samples or approved clinical use.

**Translational readiness**: Exploratory = mechanistic research only; Emerging = preclinical animal evidence or synergy with existing drugs; Early Clinical Potential = linked to patient data or close to clinical translation; Practice-adjacent = approved drug or directly relevant to RCC therapy.

At the signaling level, Cytokine signaling links ferroptosis programs to immune remodeling in RCC. CD8⁺ T-cell–derived IFN-γ activates JAK/STAT1 to repress SLC7A11/SLC3A2 (system Xc^−^), limiting cystine uptake and amplifying lipid peroxidation; IFN-γ/STAT1 also induces IRF1→ACSL4, channeling PUFAs toward ferroptosis^[99]^. Conversely, an inflammatory STAT3→GPX4 axis sustains antioxidant defense and ferroptosis resistance in RCC cells^[105]^. Transcriptomic ferroptosis signatures show enrichment of cytokine–cytokine receptor and chemokine (e.g., CXCL9/10–CXCR3) pathways, consistent with T-cell recruitment and checkpoint regulation within the TME^[106]^. These data provide the missing intermediate layers connecting metabolism to immune infiltration and PD-L1 biology in RCC.

## Multi-omics insights into therapeutic targeting of ferroptosis

RCC subtypes display distinct metabolic wiring that shapes ferroptosis thresholds and therapeutic opportunities. ccRCC accumulates polyunsaturated lipids and exhibits altered iron handling; in this background, the glutathione/GPX4 antioxidant axis becomes essential to avert lethal lipid peroxidation, aligning with preclinical hypersensitivity when system Xc^−^/SLC7A11–GPX4–FSP1 defenses are compromised^[63,101]^. (pRCC shows a different ferroptosis signature landscape, often linked to signaling programs such as MET and redox buffering, suggesting that effective ferroptosis sensitization may require concurrent constraint of cystine import and kinase pathways^[25,70]^. chRCC harbors mitochondrial/ion-transport peculiarities and emerging dependence on FSP1/CoQ10 rescue; recent data indicate context-dependent ferroptosis responses, nominating FSP1 as a rational co-target and underscoring that GPX4 or SLC7A11 inhibition alone may be insufficient in some chRCC settings^[100,103]^. Collectively, these subtype-resolved features argue for biomarker-guided enrichment – using lipid-unsaturation or iron/heme-flux readouts and SLC7A11/GPX4/FSP1 expression – and for rational combinations (e.g., TKI/mTOR/IO plus ferroptosis sensitizers) to widen the therapeutic window while respecting safety constraints.

Multi-layer sequencing has dismantled the old view of renal-cell carcinoma as a single disease, exposing a lattice of genomic, epigenomic, transcriptomic, and metabolic events that converge on ferroptosis control. Truncal lesions such as VHL or PBRM1 loss reshape iron uptake and polyunsaturated-lipid pools, while chromatin errors like SETD2 deficiency depress ferrochelatase and raise intracellular iron, lowering the threshold for lipid-peroxide collapse^[24]^. Proteogenomic mapping adds another tier: DPP9 stabilizes NRF2, restores SLC7A11 and shields tumors from sorafenib, yet DPP9 inhibition reverses this escape route^[102]^. When these omics layers are overlaid, common pressure points emerge – NRF2–SLC7A11, GPX4, and FSP1 – that together dictate ferroptosis fitness.

### From omics atlases to bedside stratification

Two streamlined mRNA signatures showcase the journey from discovery datasets to actionable diagnostics. A seven-gene score derived from TCGA stratifies overall survival and links ferroptosis susceptibility to arachidonic-acid metabolism^[107]^. Independent validation produced an eleven-gene panel enriched for immune-activation transcripts, suggesting that ferroptotic injury may amplify anti-tumor immunity^[104]^. Both assays are compatible with routine bulk RNA sequencing and already outperform pathological stage, positioning them as natural entry criteria for trials that layer ferroptosis inducers onto standard regimens.

### Targeted drugs as biochemical accelerators

Angiogenesis inhibitors – the mainstay of metastatic therapy – also modulate ferroptotic tone. Sorafenib inhibits system Xc^−^, depletes GSH, and thereby contributes to tumor regression^[108]^. Sunitinib and bevacizumab re-oxygenate myeloid compartments, boost IL-12 release, and expand cytotoxic T-cell pools, all of which favor lipid-peroxide accumulation^[109,110]^. Resistance typically emerges when STAT3 or NRF2 reactivate SLC7A11; spatial transcriptomics of post-neoadjuvant tissue confirms this switch, whereas erastin or genetic SLC7A11 silencing restores drug sensitivity^[95]^. mTOR blockade adds a second lever: everolimus suppresses 4EBP1, lowers GPX4, and magnifies erastin cytotoxicity without additional systemic burden^[111]^.

### Metabolic and hereditary niches

Metabolome-wide screens identify fumarate-hydratase-deficient tumors as cystine-addicted yet ferroptosis-resistant. Extracellular cystine depletion with cysteinase, combined with low-dose rapamycin, converts this metabolic liability into tumor regression^[112]^. At the mitochondrial interface, loss of MDH2 activates FSP1-mediated radical scavenging; re-expression of MDH2 or targeted FSP1 degradation re-sensitizes clear-cell cultures to erastin^[70]^. Lipidomics further reveals a chemerin-GPCR pathway wherein blockade of GPR1 or CMKLR1 disrupts lipid-droplet formation and provokes tandem apoptosis-ferroptosis^[73]^.

### Nanotherapeutics and stimulus-responsive delivery

Advances in delivery chemistry are widening the therapeutic window of ferroptosis inducers. A pH-responsive iron-organic framework, MIL-101(Fe)@RSL3, co-releases Fe^2^⁺and a GPX4 inhibitor within acidic lysosomes, unleashing Fenton reactions while sparing normal tissue^[76]^. Mild photothermal activation of PEG-PLGA-encapsulated Fe₃O₄nanoparticles remodels antioxidant gene expression and drives lipid peroxidation in orthotopic models with minimal off-target toxicity^[113]^. Design rules governing particle size, protein corona, and tumor permeability are now codified, accelerating translation toward first-in-human batches^[114]^.

### Low-toxicity phytochemical sensitizers

Plant-derived compounds provide inexpensive adjuncts to kinase or immune blockade. Ginsenoside Rh4 suppresses NRF2, GPX4, and catalase, thereby amplifying RSL3 lethality *in vitro* and in xenografts^[115]^. Icariside II elevates miR-324-3p to repress GPX4, whereas ursolic acid concomitantly degrades MCL-1 and SLC7A11, converting sorafenib into a dual apoptosis-ferroptosis agent^[27,116]^. Their oral bioavailability and favorable early toxicology profiles suit peri-operative or maintenance settings where intensive systemic therapy is untenable.

### Blueprint for biomarker-guided trials

A clinical scaffold is already in place: randomized first-line studies demonstrate that vascular-kinase inhibition combined with PD-1/PD-L1 blockade prolongs survival and preserves quality-of-life metrics^[117,118]^. Multi-omics offers the third pillar – patient selection. Tumors exhibiting high ferroptosis-gene scores, elevated lipid peroxides, or low FSP1 can be randomized to triplet regimens that add erastin analogues, GPX4 inhibitors, or cysteinase. Adaptive designs incorporating serial lipidomics and circulating-DNA readouts will enable real-time response tracking and early mechanistic insights.

### Subtype-specific ferroptosis vulnerabilities across RCC subtypes

ccRCC exhibits a ferroptosis-prone metabolic phenotype characterized by *VHL* loss, HIF stabilization, PUFA enrichment, and pronounced ACSL4-mediated lipid remodeling, collectively lowering the threshold for lipid peroxidation. In contrast, pRCC is marked by aberrant MET activation, which reinforces NRF2-driven antioxidant programs and SLC7A11-dependent cystine uptake, thereby establishing a ferroptosis-resistant redox state. chRCC displays even stronger ferroptosis resistance due to its reliance on the FSP1–CoQ10 lipid-antioxidant axis and reduced PUFA availability, explaining its limited sensitivity to GPX4 inhibition alone. These distinct metabolic and redox signatures underscore the need to tailor ferroptosis-targeted therapeutic strategies to RCC molecular subtypes – for example, combining MET inhibition with system Xc^−^ blockade in pRCC, or dual GPX4 and FSP1 inhibition approaches in chRCC.

## Translational and clinical applications of ferroptosis in RCC

Clinically oriented evidence suggests that ferroptosis can inform both risk stratification and therapy optimization in RCC. Ferroptosis-related gene signatures integrated with clinicopathologic variables have shown robust prognostic performance and are suitable for incorporation into pre-operative biopsies and post-operative nomograms to individualize follow-up and adjuvant decisions^[107]^. On the therapeutic front, multiple strategies sensitize RCC to ferroptosis or reverse resistance: the natural compound 2-undecanone inhibits the STAT3/GPX4 axis and amplifies sunitinib-induced lipid peroxidation and iron accumulation, nominating a rational TKI-plus-ferroptosis combination^[105]^; likewise, the mTOR inhibitor everolimus accelerates erastin/RSL3-triggered ferroptosis, supporting integration with existing targeted regimens^[111]^. Metabolic rewiring via the chemerin-GPR1/CMKLR1 axis maintains the clear-cell lipidome and buffers lipid ROS; targeting this pathway (e.g., α-NETA) reduces tumor growth and “clear-cell” morphology in patient-derived models, revealing a tractable vulnerability linked to ferroptosis susceptibility^[73]^. Precision delivery platforms (e.g., iron-co-loaded nanoparticles with GPX4 inhibition) further enhance intratumoral ferroptosis activation, although clinical translation will require standardized manufacturing and safety evaluation^[25]^. Finally, the nitric-oxide prodrug JS-K depletes GSH and down-regulates the c-Myc/GSTP1 antioxidant barrier, inducing ferroptosis and suppressing RCC xenografts – implicating GSTP1 as a dual biomarker-target for future trials^[119]^.

Given the translational promise outlined above, attention to safety and dosing windows is essential for clinical adoption. Despite promising preclinical efficacy, systemic ferroptosis induction raises non-trivial safety concerns. Conditional Gpx4 ablation in adult mice triggers hippocampal neurodegeneration and lethality, underscoring a narrow therapeutic window for GPX4 inhibition^[120,121]^. Tool GPX4 inhibitors such as RSL3 and ML162 show robust on-cell ferroptosis but have suboptimal selectivity and pharmacokinetics; importantly, recent biochemical work indicates they do not directly inhibit recombinant GPX4, suggesting off-target liabilities^[122,123]^. By contrast, cysteine depletion strategies (system x_c^−^ blockade or cyst(e)inase) can elicit tumor-selective ferroptosis *in vivo*, yet normal tissues reliant on GSH metabolism remain at risk, necessitating careful dose scheduling and monitoring^[5]^. Risk-mitigation strategies include prodrug/next-generation chemotypes with improved PK [e.g., imidazole ketone erastin (IKE)] and tumor-targeted nanodelivery (e.g., MIL-101(Fe)@RSL3), which enhance intratumoral exposure while reducing off-target toxicity^[25,124]^. Collectively, these data support advancing ferroptosis-based therapies with a safety-first design – prioritizing targeted delivery, rational combinations to enable dose-reduction, and early incorporation of toxicity biomarkers in translational studies.

## Future directions

Advancing ferroptosis toward clinical utility in RCC will hinge on integrated measurement, selection, and modulation strategies. First, spatially resolved phenotyping should be used to map “ferroptosis-vulnerable niches” by co-localizing iron flux and lipid peroxidation with antioxidant defenses (SLC7A11/GPX4/FSP1) in surgical specimens; multimodal mass-spectrometry imaging and lipidomics already resolve oxidized phospholipids *in vivo* and can be paired with pathway markers or stable-isotope tracing to avoid over-interpreting non-specific adducts^[125,126]^. Next, non-invasive enrichment and pharmacodynamic readouts are feasible with system x_c^−^ imaging: early clinical studies using [^18 F]FSPG PET demonstrate tumor-to-tumor heterogeneity in x_c^−^ activity and support dynamic monitoring before and after ferroptosis-sensitizing regimens or IO/TKI combinations^[127]^. Finally, microbiome-informed modulation merits RCC-specific testing. Intratumoral and gut microbial features correlate with prognosis and immunotherapy benefit, while metabolites act as chemical “rheostats”: bile acids can suppress ferroptosis via FXR→GPX4/FSP1 programs, whereas short-chain fatty acids (e.g., butyrate) can enhance ferroptosis sensitivity – together nominating diet, defined probiotics, or carefully controlled FMT as rational co-interventions under a safety-first design. Near-term translational studies should focus on mechanistically grounded combinations (e.g., IO/TKI with cyst(e)ine-restriction or x_c^−^ blockade, FSP1 co-targeting in chRCC-like contexts) and embed longitudinal toxicity/PD monitoring; preclinical work with cyst(e)inase, imidazole-ketone erastin, and delivery approaches provides actionable scaffolds while medicinal-chemistry barriers to direct GPX4 inhibition are addressed. Synchronizing sensitizers with immune activation is also mechanistically justified, as IFN-γ–activated CD8⁺ T cells can drive tumor ferroptosis, albeit with attention to timing to avoid T-cell ferroptosis.

## Conclusion

Tables 2 and 3 summarize the multi-omics characteristics of ferroptosis in RCC. Genomic surveys show that loss of *VHL* and copy number gains in SLC7A11 and FSP1 enlarge the pool of ferrous iron while strengthening the xCT–GPX4–FSP1 antioxidant axis, an adaptation that helps tumor cells survive oxidative stress^[10]^. Layered transcriptomic and proteomic maps confirm this paradox: lipid-peroxidation engines such as ACSL4 decline in aggressive disease, yet antioxidant nodes rise, producing a metabolically poised but guarded phenotype^[25]^. Metabolomic profiles deepen the picture by revealing large stores of cholesterol and ether lipids that blunt lipid-oxidative injury until GPX4 is disabled, at which point these reservoirs become toxic^[76]^. Integrative machine-learning models that merge these omics layers already discriminate distinct ferroptosis programmes, each with characteristic immune infiltrates and drug sensitivities, suggesting immediate opportunities for patient stratification^[128]^.

**Table 2:** Prognostic and diagnostic models of ferroptosis in RCC.

Omic layer	Key ferroptosis-related molecular/omics alterations in RCC	Diagnostic / prognostic biomarkers	Evidence level	Key reference (Author and Year)
Genomics	ccRCC cells show VHL loss ➜ HIF-2α–driven GPX4/ACSL4-dependence; recurrent copy-loss of GPX4 locus in papillary RCC; fumarate-hydratase (FH) deficiency sensitizes to cyst(e)inase-rapamycin ferroptosis	FH-mut, GPX4 CNV score, and 13-gene FR-risk panel	Preclinical	^[12–20,59,103]^
Epigenomics	Promoter hyper-methylation of SLC7A11 (xCT) lowers cystine import; miR-324-3p/miR-214-3p up-regulate ferroptosis by GPX4 silencing; HO-1 up-regulation (histone-acetylated) augments luteolin-triggered ferroptosis	Methyl-SLC7A11 index, miR-324-3p levels	*In vitro*/Preclinical	^[21–27,52,110]^
Proteomics	Quantitative MS highlights elevated ACSL4, TRIB3, CX3CL1 and depressed GSTP1, GPX4 in aggressive ccRCC; riproximin-induced lipid-ROS network captured in phosphoproteome	ACSL4/TRIB3 protein ratio predicts poor OS; GSTP1 low level = ferroptosis-susceptible subset	Clinical/*in vitro*	^[16,49–58]^
Transcriptomics and Single-cell-omics	Bulk RNA-seq: 8- to 15-gene ferroptosis signatures (ACSL1, POLD1, and CX3CL1…); scRNA-seq identifies TRIB3^high epithelial clones and xCT^low CD8^ + niches; exosomal 13-gene model (Wang) yields AUC ≈ 0.96	13-gene exosome panel (Wang 2023); 6-lncRNA FR-signature (Ju 2022); TRIB3^high cluster load	Clinical	^[28–48]^
Metabolomics	CC-RCC accumulates cholesterol and ether-lipids that quench lipid-ROS; GPR1/CMKLR1 rewires TAG oxidation; ACSL8/11 and ACOT8/11 act as metabolic ferroptosis nodes; corosolic-acid triggers non-apoptotic lipid-ROS death	Elevated free-cholesterol (Zhao 2023); ACOT11 over-expression = worse DFS	Preclinical	^[53,59–73]^
Tumor micro-environment and Immunity	CX3CL1 from tumor cells increases CD8^ + T-cell recruitment and ferroptosis sensitivity; CD8-T-cell IFN-γ down-regulates xCT; M2-TAM-derived TRIB3 confers resistance; exosomal GAG-mediated lipoprotein uptake protects from ferroptosis	CX3CL1 high = better ICI response; TRIB3-M2 signature = immune-cold	Preclinical/Clinical	^[69,72,89–95]^

Biomarkers have been validated in at least one patient cohort or animal model.

**Evidence Level**: *In vitro* = cell-based experiments only; Preclinical (*in vivo*) = supported by animal models; Clinical = validated in patient samples or approved clinical use.

**Table 3:** Therapeutic strategies of ferroptosis in RCC.

Omic layer	Key ferroptosis-related molecular/omics alterations in RCC	Potential therapeutic targets/strategies	Evidence level	Translational readiness	Key reference (Author and Year)
Genomics	ccRCC cells show VHL loss ➜ HIF-2α–driven GPX4/ACSL4-dependence; recurrent copy-loss of GPX4 locus in papillary RCC; fumarate-hydratase (FH) deficiency sensitizes to cyst(e)inase-rapamycin ferroptosis	HIF-2α antagonists + GPX4 inhibitors; cyst(e)inase–rapamycin combo	Preclinical (xeno)	Emerg.	^[12–20,59,103]^
Epigenomics	Promoter hyper-methylation of SLC7A11 (xCT) lowers cystine import; miR-324-3p/miR-214-3p up-regulate ferroptosis by GPX4 silencing; HO-1 up-regulation (histone-acetylated) augments luteolin-triggered ferroptosis	DNMT inhibitors to de-repress xCT; BET-inhibitor + HO-1 axis modulation	*In vitro*/Preclinical (cell + xeno)	Expl.	^[21–27,52,110]^
Proteomics	Quantitative MS highlights elevated ACSL4, TRIB3, CX3CL1 and depressed GSTP1, GPX4 in aggressive ccRCC; riproximin-induced lipid-ROS network captured in phosphoproteome	GSTP1 inhibitors; TRIB3 blockade combined with TKI	Clinical + *In vitro*	Early Clin.	^[16,49–58]^
Transcriptomics and Single-cell-omics	Bulk RNA-seq: 8- to 15-gene ferroptosis signatures (ACSL1, POLD1, and CX3CL1…); scRNA-seq identifies TRIB3^high epithelial clones and xCT^low CD8^ + niches; exosomal 13-gene model (Wang) yields AUC ≈ 0.96	RNA-interference vs. TRIB3; IFN-γ/STAT1 to down-regulate xCT in CD8-rich tumors	Clinical	Early Clin.	^[28–48]^
Metabolomics	CC-RCC accumulates cholesterol and ether-lipids that quench lipid-ROS; GPR1/CMKLR1 rewires TAG oxidation; ACSL8/11 and ACOT8/11 act as metabolic ferroptosis nodes; corosolic-acid triggers non-apoptotic lipid-ROS death	Statin + GPX4 inhibitor; chemerin-GPR1 blockade; corosolic-acid analogues	Preclinical	Emerg.	^[53,59–73]^
Tumor micro-environment and Immunity	CX3CL1 from tumor cells increases CD8^ + T-cell recruitment and ferroptosis sensitivity; CD8-T-cell IFN-γ down-regulates xCT; M2-TAM-derived TRIB3 confers resistance; exosomal GAG-mediated lipoprotein uptake protects from ferroptosis	Combine anti-PD-1 with xCT inhibitors; TAM re-programming (anti-TRIB3) + lipid peroxidation amplifiers	Preclinical + Clinical correlations	Early Clin.	^[69,72,89–95]^

Biomarkers have been validated in at least one patient cohort or animal model.

cell = *in vitro*; xeno = mouse xenograft (preclinical *in vivo*); clin = clinical/patient data.

Evidence Level: *In vitro* = cell-based experiments only; Preclinical (*in vivo*) = supported by animal models; Clinical = validated in patient samples or approved clinical use.

Translational Readiness: Expl. = Exploratory (mechanistic research only); Emerg. = Emerging (preclinical animal evidence or synergy with existing drugs); Early Clin. = Early Clinical Potential (linked to patient data or close to clinical translation); Pract.-adj. = Practice-adjacent (approved drug or directly relevant to RCC therapy).

Future progress rests on turning this delicate equilibrium toward cell death. Rational combination therapy is one avenue: pairing GPX4 or glutaminase inhibitors with DNA-damage agents or immune checkpoint blockade has produced durable tumor regressions in *VHL*-deficient models^[10]^. Nanotechnology offers another strategy. A tumor-responsive iron–organic framework that co-releases ferrous ions and RSL3 unleashes a cascading burst of lipid peroxidation, eradicating orthotopic RCC while sparing normal tissue^[76]^. Artificial-intelligence tools are accelerating translation. Deep-learning platforms integrate bulk, single-cell, and plasma-derived omics to rank patients by ferroptosis vulnerability and predict optimal drug pairs with clinical-grade accuracy. Convolutional networks now connect routine CT scans to molecular ferroptosis signatures and survival, making non-invasive monitoring feasible^[129]^. Large-language-model pipelines are also being explored to transform complex multiomics outputs into eligibility criteria for adaptive trials^[130]^. Prospective studies that embed spatial-omics readouts, dynamic metabolomic monitoring, and AI-guided enrolment will be critical. If executed rigorously, precision ferroptosis therapy could convert the metabolic liabilities of RCC into lasting clinical benefit.

## Data Availability

Original data generated and analyzed during this study are included in this publication.
